# Xenon preconditioning: molecular mechanisms and biological effects

**DOI:** 10.1186/2045-9912-3-3

**Published:** 2013-01-10

**Authors:** Wenwu Liu, Ying Liu, Han Chen, Kan Liu, Hengyi Tao, Xuejun Sun

**Affiliations:** 1 Department of Diving Medicine, Secondary Medical University, No 800 Xiangyin Road, Yangpu District, Shanghai 200433, People’s Republic of China; 2 Department of General Surgery, 411 Hospital, No 15 Dongjiangwan Road, Hongkou District, Shanghai, 200081, People’s Republic of China; 3 Institute of Nautical Medicine, Nantong University, Jiangsu, 226019, People’s Republic of China; 4 Department of Pathology, Yantaishan Hospital, Yantai, Shandong, 264000, People’s Republic of China

**Keywords:** Xenon, Preconditioning, Neuroprotection, Cardioprotection, Mechanism

## Abstract

Xenon is one of noble gases and has been recognized as an anesthetic for more than 50 years. Xenon possesses many of the characteristics of an ideal anesthetic, but it is not widely applied in clinical practice mainly because of its high cost. In recent years, numerous studies have demonstrated that xenon as an anesthetic can exert neuroprotective and cardioprotective effects in different models. Moreover, xenon has been applied in the preconditioning, and the neuroprotective and cardioprotective effects of xenon preconditioning have been investigated in a lot of studies in which some mechanisms related to these protections are proposed. In this review, we summarized these mechanisms and the biological effects of xenon preconditioning.

## 

The noble gases are a group of chemical elements with very similar properties: they are all odorless, colorless, monatomic gases with very low chemical reactivity under standard conditions. The six noble gases occurring naturally are helium (He), neon (Ne), argon (Ar), krypton (Kr), xenon (Xe), and the radioactive radon (Rn) [1]. Among these noble gases, xenon is the most frequently investigated and widely applied in medicine. Xenon has been used as an anesthetic [2], to treat brain and heart injuries due to its neuroprotection and cardioprotection [3,4] and in single photon emission computed tomography (SPECT) [5].

Preconditioning is a process by which an organism’s exposure to a stress/stimulus permits it to decrease cellular damage or death when exposed to a subsequent greater or more sustained stress. To date, a large number of strategies have been developed for preconditioning such as lipopolysaccharide, heat and seizure, hypoxia and hyperoxia [6]. In this brief review, we introduce the protective effects of xenon preconditioning, not the xenon treatment.

## Introduction of xenon

Xenon is a colorless, heavy, odorless noble gas and was discovered by William Ramsay and Morris Travers in 1898. Xenon derives its name from the Greek word for “stranger” [7]. Xenon is a trace gas in Earth’s atmosphere and much more expensive than the lighter noble gases due to its very low concentration in air (0.5 ppm) [2]. It is inert to most common chemical reactions because the outer valence shell contains eight electrons.

To date, xenon has been commercially used for lasers, high intensity lamps, flash bulbs, jet propellant in the aerospace industry, X-ray tubes, and in medicine [7].

In medicine, xenon has been used experimentally in clinical anesthetic practice for more than 50 years. As a general anesthetic, xenon possesses many advantages. For example, its blood-gas partition coefficient is extremely small (0.115), which results in a rapid onset and offset of its action. It lacks teratogenicity and can produce profound analgesia which thereby inhibits the surgery induced hemodynamic and catecholamine responses. It is also a potent hypnotic and does not produce hemodynamic depression because it at least in part has no influence on some important ion channels [2,7]. In addition, it has been confirmed that xenon can confer neuroprotective [3] and cardioprotective [4,8] effects. Unlike nitrous oxide (N_2_O), xenon is not a greenhouse gas and so it is also viewed as environmentally friendly [9]. Xenon that is vented into the atmosphere is being returned to its original source, and thus environmental pollution is unlikely. However, its relatively high cost has precluded its more widespread clinical use.

## Mechanisms underlying bioeffects of xenon

Numerous studies have been conducted to investigate the mechanisms of xenon’s bioeffects. Xenon can potently inhibit the N-methyl-D-aspartate (NMDA) receptors non-competitively, with little effect on the γ-aminobutyric acid A (GABA_A_) receptor and non-NMDA glutamatergic receptor [10]. In the study of Yamakura and Harris, their results showed N_2_O (0.58 atmosphere [atm]) and xenon (0.46 atm) exhibited similar effects on various receptors, and the NMDA receptors, and nACh receptors composed of β2 subunits are likely targets for NO and xenon [11]. In addition, xenon (and NO) has been reported to competitively inhibit the 5-hydroxytryptamine receptor 3A (5HT3A receptor) expressed in the xenopus oocytes [12] but this has yet to be confirmed in mammalian cells.

In addition, the xenon induced anesthesia is related to the inhibition of the calcium ATPase pump on the cell membrane of synapses [13], which results from a conformational change when xenon binds to nonpolar sites inside the protein [14], and the non-specific interactions between the xenon and the lipid membrane [15].

## Mechanisms of protective effects of xenon preconditioning

### Cardioprotection, neuroprotection, renoprotective and endothelial protection

In 2005, a German group found that exposure to 70% xenon 45 min before myocardial ischemia could confer cardioprotection in a rat model, in which the activation of ε isoform of protein kinase C and its downstream target p38 mitogen-activated protein kinase (MAPK) is a central molecular mechanism [16]. Eight months later, the same group preconditioned rats with same methods, but hearts without experiencing ischemia/reperfusion were collected for detection. Their results demonstrated that xenon preconditioning enhanced the translocation of heat shock protein 27 (HSP27) to the particulate fraction and increased F-actin polymerization and activated MAPKAPK-2 and HSP27 downstream of PKC and p38 MAPK. Moreover, F-actin and pHSP27 were colocalized after xenon preconditioning. Their findings link xenon preconditioning to the cytoskeleton, revealing new insights into the mechanisms of xenon preconditioning *in vivo*[17]. In another study of Weber et al., they found xenon preconditioning increased the translocation of PKC-ε to membrane regions, and the mitochondrial adenosine triphosphate (ATP) dependent potassium (K_ATP_) channels and 3^′^ phosphatidylinositol-dependent kinase-1 (PDK-1) located upstream of PKC-ε and were crucial for the activation of PKC-ε [18]. In addition, they noted ERK 1/2, but not JNK, was also a mediator of xenon preconditioning [19]. A group in the USA found the cardioprotection of xenon preconditioning was attributed to the phosphorylation of Akt, glycogen synthase kinase 3 β (GSK-3β), preservation of mitochondrial function, and inhibition of Ca^2+^-induced mitochondrial permeability transition (MPT) pore opening (Table 1) [20].

**Table 1:** Mechanisms of protective effects of xenon preconditioning

**Mechanisms underlying protective effect of xenon preconditioning**	**Organ protection**
Activation of PKC-ε isoform and p38 MAPK [16]	Cardioprotection
HSP27 translocation, F-actin polymerization, activation of MAPKAPK-2, PKC and p38 MAPK [17]	Cardioprotection
PKC-ε translocation, mitochondrial ATP dependent K_ATP_ channels, PDK-1 [18]	Cardioprotection
ERK 1/2 [19]	Cardioprotection
Phosphorylation of Akt and GSK-3β, preservation of mitochondrial function, and inhibition of Ca^2+^-induced MPT pore opening [20]	Cardioprotection
ADNP transcription [21]	Neuroprotection
Opening of plasmalemmal K_ATP_ channels [22]	Neuroprotection
Plasma IL-1β reduction and hippocampal HSP72 increase [23]	Neuroprotection
HIF-1α, EPO and VEGF [24-26]	Renoprotection
Reduction in ICAM-1, VCAM-1 and NF-κB [27]	Protection on endothelial cells
COX-2 activity [32]	Cardioprotection
Enhanced phosphorylated cyclic adenosine monophosphate response element binding protein signaling [33]	Neuroprotection
pCREB-regulated synthesis of proteins [34]	Neuroprotection

The neuroprotection of xenon preconditioning was attributed to the transcription of activity-dependent neuroprotective protein (ADNP) in neonatal rats [21], the opening of plasmalemmal K_ATP_ channels in neuronal-glial cocultures [22] and a reduction in the plasma IL-1β and an up-regulation of hippocampal HSP72 in the surgery and/or isoflurane induced postoperative cognitive decline (POCD) model [23]. Moreover, the neuroprotection of xenon preconditioning was independent of gender in mouse transient middle cerebral artery occlusion model [24].

In the study of Ma et al., the xenon preconditioning was found to be a natural inducer of hypoxia-inducible factor (HIF-1α) [24,25]. Xenon preconditioning can activate HIF-1α and its downstream effectors erythropoietin (EPO) and vascular endothelial growth factor (VEGF) in a time-dependent manner in the kidneys *in vivo* and *in vitro*[25,26].

In the human umbilical vein endothelial cells, xenon preconditioning was found to prevent tumor necrosis factor-α (TNF-α) induced mRNA and protein expression of intracellular cell adhesion molecule 1 (ICAM-1) and vascular cell adhesion molecule 1 (VCAM-1) and decreased the TNF-α induced transcriptional activity of nuclear factor κB (NF-κB), but had no effect on the TNF-α induced E-selectin expression [27].

### Contribution of anesthetic property of xenon to protective effects

Studies also revealed that preconditioning with other noble gases without anesthetic properties could exert protective effect [28,29]. These findings supported the contention that the protection of preconditioning with noble gases was independent of the anesthetic properties *per se*. However, the protective effect of preconditioning with other noble gases was not found in the human tubular kidney cells, and even helium by comparison significantly enhanced the cell injury [26]. Additionally, in a study of Baumert et al., they did not confirm the cardioprotective effect of brief, intermittent xenon preconditioning, but the xenon anesthesia (xenon 70%, continued before and after myocardial ischemia) exert protective effect on myocardial ischemia [30]. Moreover, the xenon exposure induced gene expression profile was also found to be different from that following N_2_O (another anesthetic) exposure [31].

### Late protective effect of xenon preconditioning

In previous studies, pharmacological preconditioning not only produces early protection but induces late protective effect. This was true in the xenon preconditioning [24,32-34]. The late myocardial protective effect of xenon preconditioning was found to be closely related to the cyclooxygenase-2 (COX-2) activity because inhibition of COX-2 abolished this cardioprotective effect and the mRNA and protein expression of COX-2 remained unchanged following xenon preconditioning [32], the enhanced phosphorylated cyclic adenosine monophosphate response element binding protein signaling [33] and the phosphorylated cAMP-response element binding protein (pCREB)-regulated synthesis of proteins that promote survival against neuronal injury (Table 1) [33,34].

### Comparisons of xenon preconditioning with other strategies for preconditioning

Investigators also compared the protective effects of xenon preconditioning with those of ischemia, anesthetic(s) and hypoxia preconditioning. In the experimental myocardial infarction model, results showed combined isoflurane/xenon preconditioning reduced infarct size but not more than isoflurane alone. Ischemic preconditioning was more effective than the anesthetics [35]. In the rat N_2_O- and isoflurane-induced neuroapoptosis model, xenon preconditioning was found to prevent N_2_O oxide- and isoflurane-induced neuroapoptosis (*in vivo* and *in vitro*) and cognitive deterioration (*in vivo*). However, N_2_O - and isoflurane-induced neuroapoptosis was exacerbated by hypoxic pretreatment. N_2_O pretreatment had no effect [36]. In the study of Baumert et al., myocardial infarct size was reduced by ischemic preconditioning but less so by xenon anesthesia, and brief, intermittent exposure to xenon before myocardial ischemia did not reduce myocardial infarct size [30]. However, in the study of Weber et al., ischemic preconditioning induced by 3 × 5 min coronary artery occlusion reduced infarct size to a similar extent like anesthetic induced preconditioning [16].

## Conclusion and perspectives

Although the anesthetic properties of xenon have been known for more than 50 years and the neuroprotection and cardioprotection of xenon demonstrated for more than 10 years, xenon preconditioning is still in its infant stage. The neuroprotective and cardioprotective effects of xenon preconditioning have been confirmed in the majority of studies, but clinical studies have not been reported. In addition, numerous signaling pathways and a large amount of molecules have been shown to involve in the protective effects of xenon preconditioning, but the relative contribution of each pathway or molecule is unclear to the protective effects of xenon preconditioning.

As shown above, the cost of xenon for anesthesia and treatment is high due to the large amount of xenon used, which is the major factor limiting the wide application of xenon. However, in the available experiments on xenon preconditioning, xenon was used in combination with oxygen at a ratio of 7:3 (v/v), and preconditioning was done with 3 cycles of xenon/oxygen administered for 5 min periods [20] or for up to 20 min [23]. If the protection of this strategy is confirmed, the cost of xenon may be significantly reduced as compared to that in the anesthesia and treatment. With the development of technology, closed-circuit xenon delivery is introduced. In the study of Chakkarapani et al., they reported that the xenon consumption was minimal (<$2/h at $10/L) when the gas exchange was maintained [37]. Thus, xenon preconditioning is very cost-effective. Furthermore, xenon is not flammable and can be easily applied at bedside. Thus, we postulate that xenon preconditioning may become an alternative strategy for the prevention of diseases or injuries.

## Competing interests

The authors declare that they have no competing interests.

## Authors’ contributions

Tao HY and Sun XJ outlined this review; Liu WW, Liu Y, Chen H and Liu K searched the database and summarized the findings; Liu WW and Liu Y drafted this manuscript; Tao HY and Sun XJ revised this review. All authors read and approved the final manuscript
